# Supplementary material for: SALM1 controls synapse development by promoting F‐actin/PIP2‐dependent Neurexin clustering
Source: EMBO J. 2019 Aug 1;38(17):e101289. doi: 10.15252/embj.2018101289 (PMC6717895; doi:10.15252/embj.2018101289)
Supplement: Supplementary file 1 — Appendix [file EMBJ-38-e101289-s001.pdf]

Appendix

Table of Contents

Figure S1. SALM1 localizes to pre- and postsynaptic membranes. 2

Figure S2. Efficient knockdown of SALM1 using shRNA's. 3

Figure S3. SALM1 is enriched at synapses in DIV9 mouse excitatory hippocampal neurons. 4

Figure S4. PIP2 and F-actin are enriched at SALM1 puncta. 5

Figure S5. SALM1 clusters Neurexin via F-actin/PIP2 and is independent on SALM1's PDZ domain. 6-7

Figure S6. SALMs contain a highly conserved polybasic region and can all cluster Nrxn1β. 8

Figure S7. SALM1 depletion does not affect neurite morphology, but impairs synapse development and synaptic vesicle recruitment. 9-10

Figure S8. SALM1 depletion does not affect synaptic transmission during late developmental stage (DIV9→16). 11

References. 12

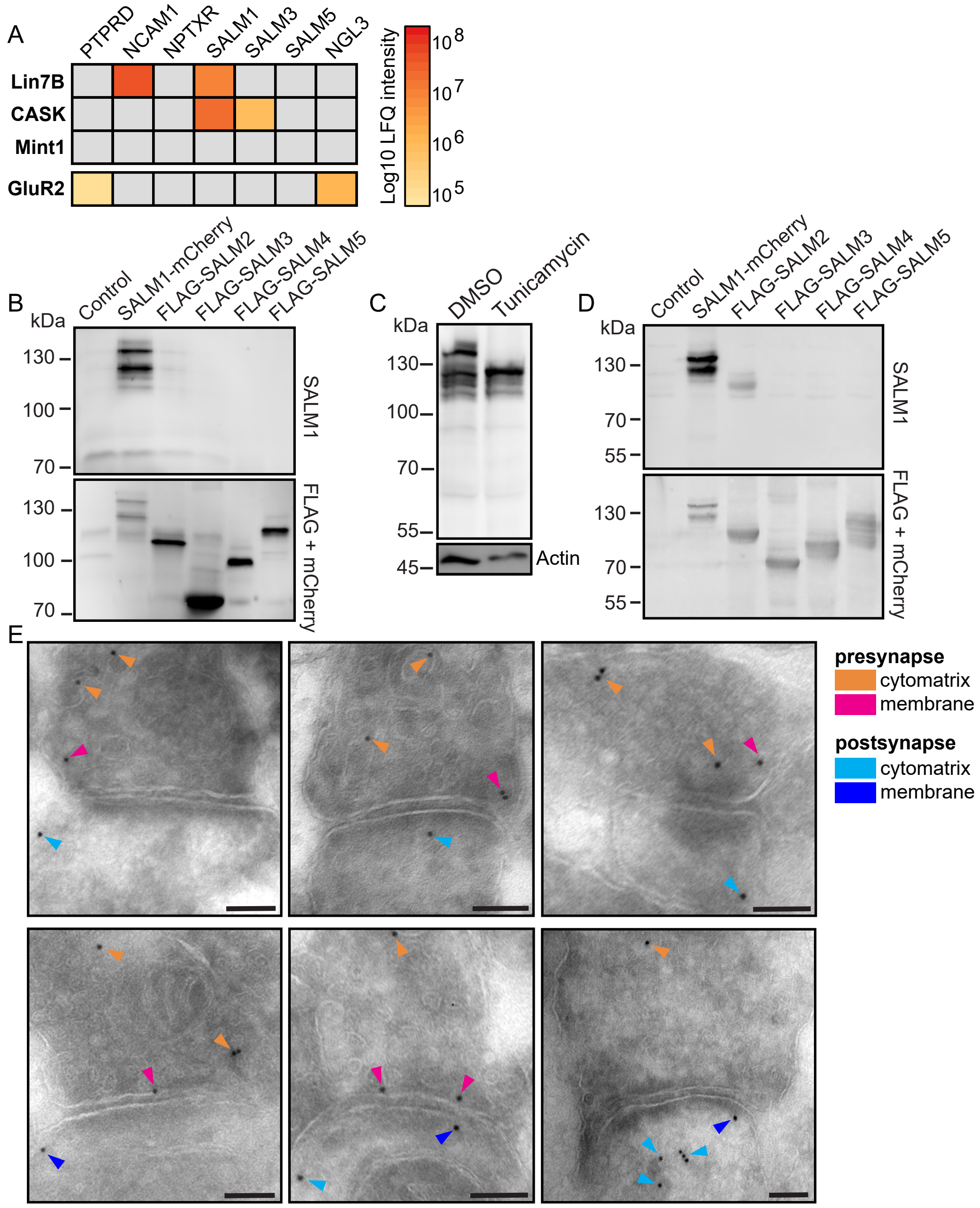

Figure S1. SALM1 localizes to pre- and postsynaptic membranes (related to figures 1 and 2).

A) Table showing all tested synaptic adhesion molecules in a proteomics screen using CASK, Mint1, Lin7b or GluR2 as bait. Values represent the average log10 LFQ intensity of 3 pull down replicates.

B) Western blot showing lysates of HEK cells transfected with empty vector (control), SALM1-mCherry or SALM2, 3, 4 or 5-FLAG stained for SALM1. The SALM1 antibody (SySy) specifically recognized SALM1-mCherry. FLAG and mCherry stainings were used to indicate loading of SALM proteins.

C) Western blot showing lysates of HEK cells transfected with SALM1-mCherry and treated with DMSO or Tunicamycin. Actin staining was used as loading control.

D) Western blot showing lysates of HEK cells transfected with empty vector (control), SALM1-mCherry or SALM2, 3, 4 or 5-FLAG stained for SALM1. The SALM1 antibody (ITK) recognized SALM1-mCherry with high specificity. FLAG and mCherry stainings were used to indicate loading of SALM proteins.

E) Electron micrographs of hippocampal slices stained for endogenous SALM1 with immuno gold labeling. SALM1 immunogold particles are detected in presynapses (orange and magenta arrows) and postsynapses (cyan and blue arrows). Bars = 100nm.

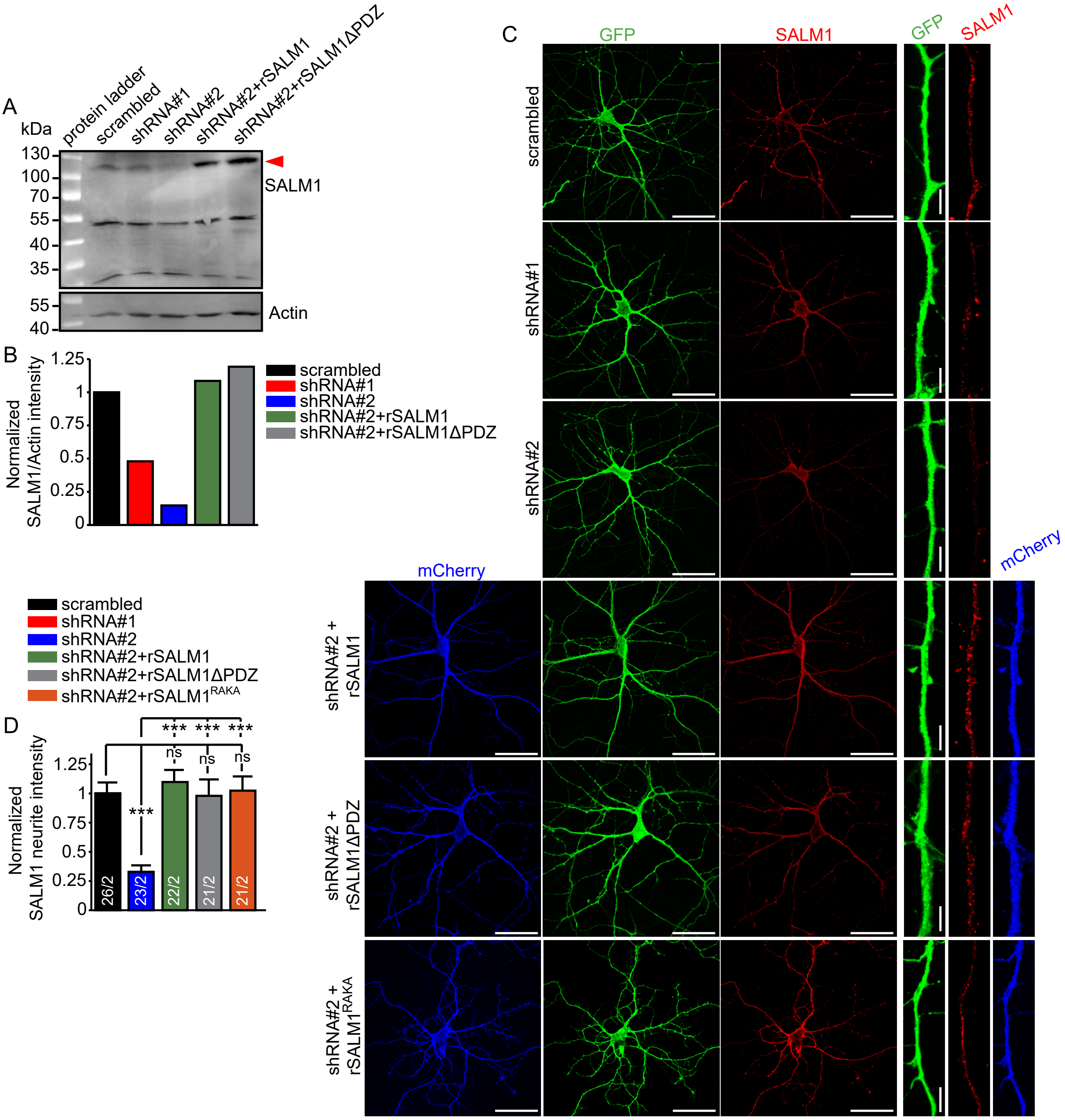

Figure S2. Efficient knockdown of SALM1 using shRNA's (related to figure 2 and 3).

A) Western blot of DIV14 mouse cortical primary mass cultures infected with scrambled, shRNA#1, shRNA#2, shRNA#2+rSALM1 or shRNA#2+SALM1ΔPDZ at DIV7. Actin was used to control for protein load. Red arrowhead indicates the specific SALM1 band.

B) Quantification of the expression of SALM1 observed on western blot (D) normalized to the expression levels of actin.

C) Immunocytochemistry using a SALM1 specific antibody on sandwich cultured mouse hippocampal neurons infected at DIV3 with the indicated lentiviruses and analyzed at DIV10. Neurons were stained for total SALM1 levels. GFP was used as indicator of successful infection of scrambled, shRNA#1 or shRNA#2 viruses. mCherry was used as indicator of successful infection of rSALM1, rSALM1RAKA or rSALM1ΔPDZ viruses. Bars=20μm in full neuron images. Bars=5μm in zoomed images.

D) Average normalized neurite SALM1 intensity ± SEM per neuron. Numbers in bars indicate total number of cells / total number of independent experiments. A Kruskal-Wallis test with posthoc paired comparisons was used ($p < 0.001$). ns = not significant and *** = $p < 0.001$.

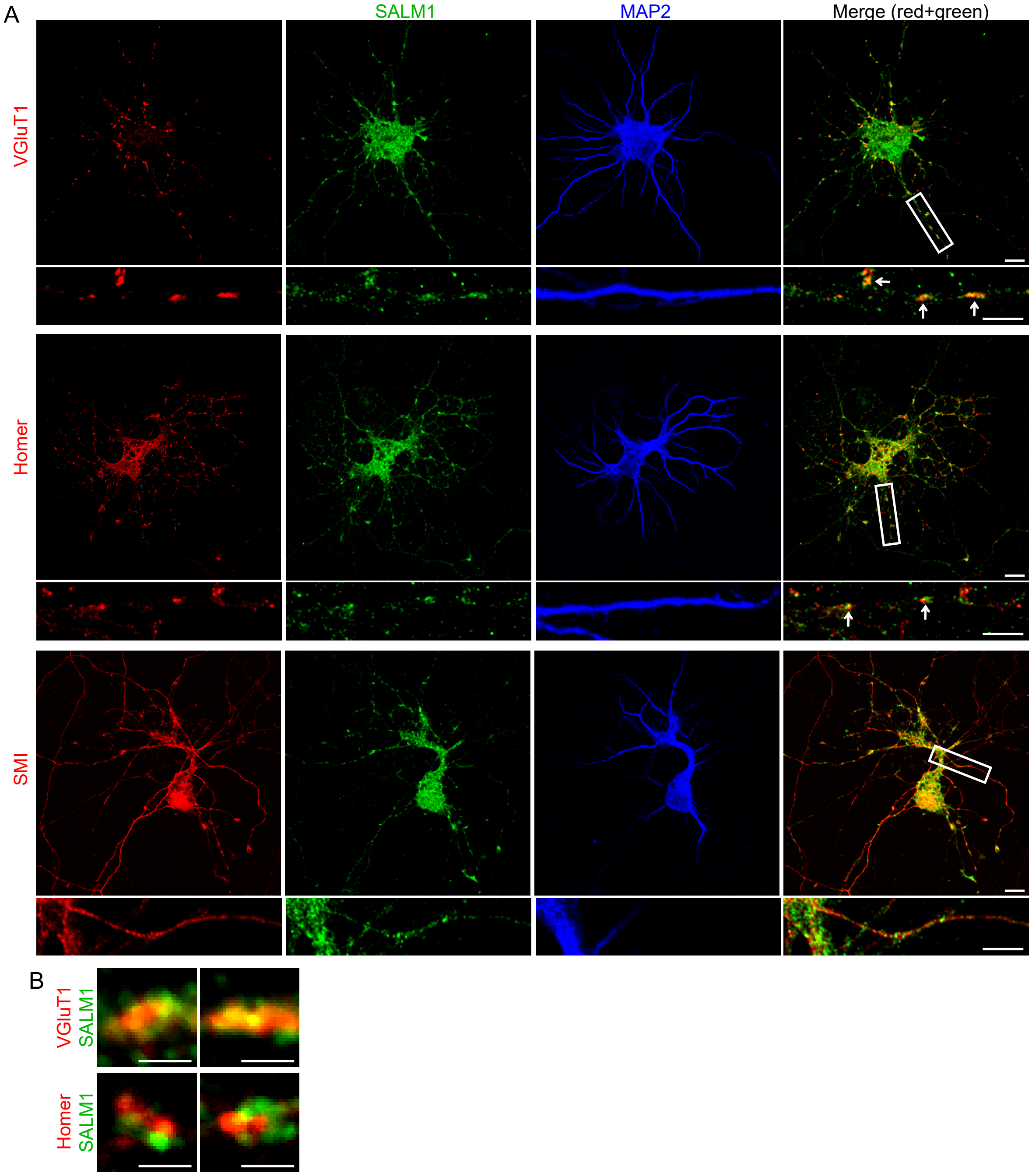

Figure S3. SALM1 is enriched at synapses in DIV9 mouse excitatory hippocampal neurons (related to figure 2).

A) Sandwich cultured mouse hippocampal neurons stained at DIV9 for total endogenous SALM1 (green), dendritic marker MAP2 (blue) and synapse markers Homer or VGluT1 (red), or the axonal marker SMI-312 (red). Boxes indicate area of zoom. Arrows indicate overlap between SALM1 and synapse markers. Bars = 10μm in full neuron images. Bars = 5μm in zoomed images.

B) Example images showing differential overlap of SALM1 with Homer or VGluT1. Bars = 1μm.

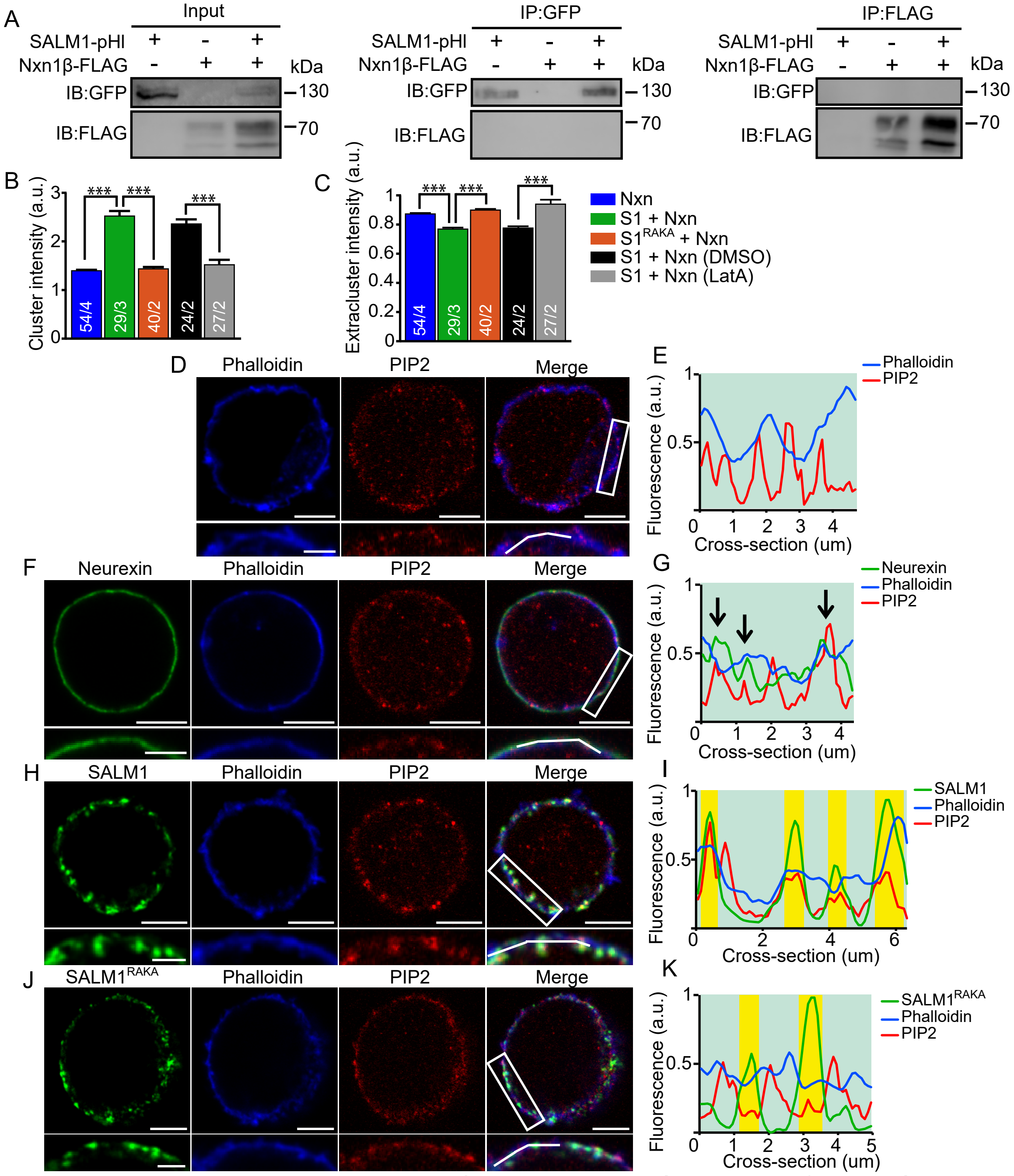

Figure S4. PIP2 and F-actin are enriched at SALM1 puncta (related to figure 5).

A) Co-immunoprecipitation blot of HEK cells expressing SALM1-pHl, Nrxn1β-FLAG or both constructs. Samples were pulled down using GFP antibody (IP:GFP) or FLAG antibody (IP:FLAG).

B) Average surface Nrxn1β-FLAG cluster intensity ± SEM on HEK cells expressing the indicated different constructs.

C) Average surface Nrxn1β-FLAG intensity ± SEM detected outside clusters on HEK cells expressing the indicated different constructs.

The n is indicated in the bars and represent the total number of cells/total number of independent cultures. S1=SALM1-pHl, Nxn= Nrxn1β-FLAG and S1RAKA=SALM1RAKA-pHl. Kruskal-Wallis tests with post hoc paired comparisons were used on B ($p < 0.001$) and C ($p < 0.001$). *** = $p < 0.001$.

D, F, H and J) Single z-slice images through wild type HEK cells (D) or HEK cells expressing either Nrxn1β-FLAG (F), SALM1-pHl (H) or SALM1RAKA-pHl (J) and stained for Phalloidin (blue), PIP2 (red) and GFP or FLAG (green, surface staining). White boxes indicate area of zoom, zoomed images are depicted below the full image for each channel. Bars=5μm in full HEK cell images. Bars = 2μm in zoomed images.

E, G, I and K) Representative fluorescence intensity plots of cross-sections depicted by white lines in the zoomed merge images in D, F, H and J. Black arrows in F highlight Neurexin/PIP2 co-enrichment. SALM1 and SALM1RAKA puncta are highlighted in yellow.

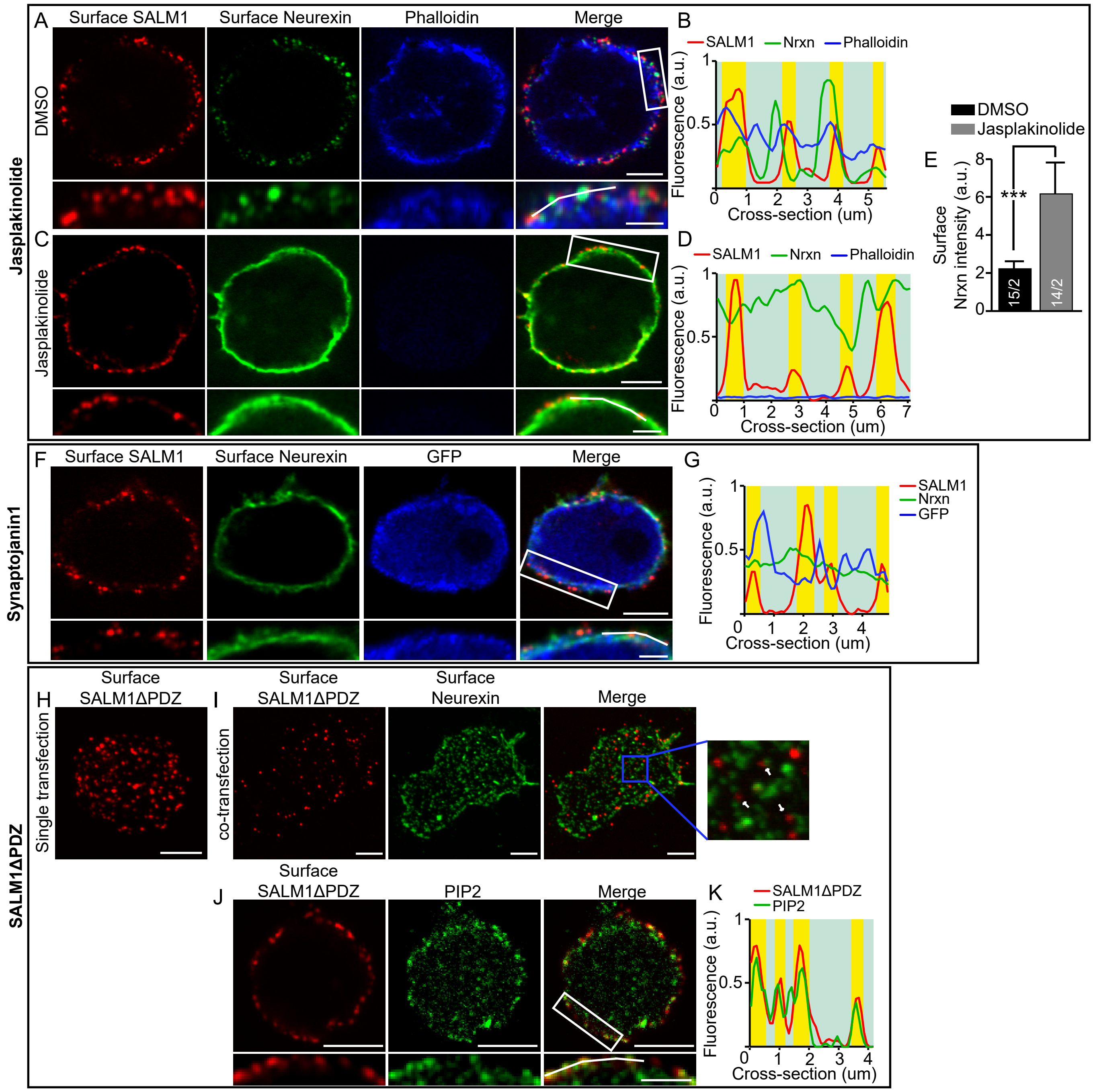

Figure S5. SALM1 clusters Neurexin via F-actin/PIP2 and is independent on SALM1's PDZ domain (related to figure 5).

A & C) Example of surface expression patterns of SALM1-pHI (red) and Nrxn (green) when co-expressed in HEK cells treated with DMSO (A) or Jasplakinolide (B). Phalloidin (blue) was used to stain for F-actin. Lack of F-actin staining by Phalloidin was used as indication of effective Jasplakinolide treatment since Phalloidin and Jasplakinolide compete for the same binding site on F-actin (Bubb et al., 1994). Images represent single z-slices. White boxes indicate area of zoom, zoomed images are depicted below the full image for each channel. Bars=5µm in full HEK cell images. Bars = 2µm in zoomed images.

B & D) Representative fluorescence intensity plots of the cross-section depicted by the white line in the zoomed merge images in A and C. SALM1 puncta are highlighted in yellow.

E) Average surface Nrxn1β-FLAG intensity ± SEM per HEK cell for SALM1-pHI / Nrxn1β-FLAG co-expressing cells treated with DMSO or Jasplakinolide. The numbers given at the base of the bars indicate the total number of HEK cells / the total number of independent cultures. A T-test was used to test for significance (*** $p < 0.001$).

F to K are described on page 7.

Figure S5. Continued

F) Example of surface expression patterns of SALM1 (red) and Nrxn (green) when co-expressed in HEK cells together with membrane targeted Synaptojanin1. GFP staining (blue) was used as marker for expression of the Synaptojanin1IRES2GFP construct (see also Methods). Images represent single z-slices. White boxes indicate area of zoom, zoomed images are depicted below the full image for each channel. Bars=5μm in full HEK cell images. Bars = 2μm in zoomed images.

G) Representative fluorescence intensity plot of the cross-section depicted by the white line in the zoomed merge image in A. SALM1 puncta are highlighted in yellow.

H) Example of expression pattern of SALM1ΔPDZ-pHl at the surface of a HEK cell. Image represents a collapsed z-stack. Bar=5μm

I) Example of surface expression patterns of SALM1ΔPDZ-pHl (red) and Nrxn1β-FLAG (green) when co-expressed in HEK cells. Images represent collapsed z-stacks. Blue box indicates area of zoom, white arrows indicate examples of SALM1ΔPDZ puncta in close proximity of Nrxn1β puncta. Bars=5μm in full HEK cell images. Bars = 2μm in zoomed images.

J) Example of surface expression patterns of SALM1ΔPDZ-pHl (red) and PIP2 (green) when co-expressed in HEK cells. Images represent single z-slices. White boxes indicate area of zoom, zoomed images are depicted below the full image for each channel. Bars=5μm in full HEK cell images. Bars = 2μm in zoomed images.

K) Representative fluorescence intensity plot of the cross-section depicted by the white line in the zoomed merge image in C. SALM1ΔPDZ puncta are highlighted in yellow.

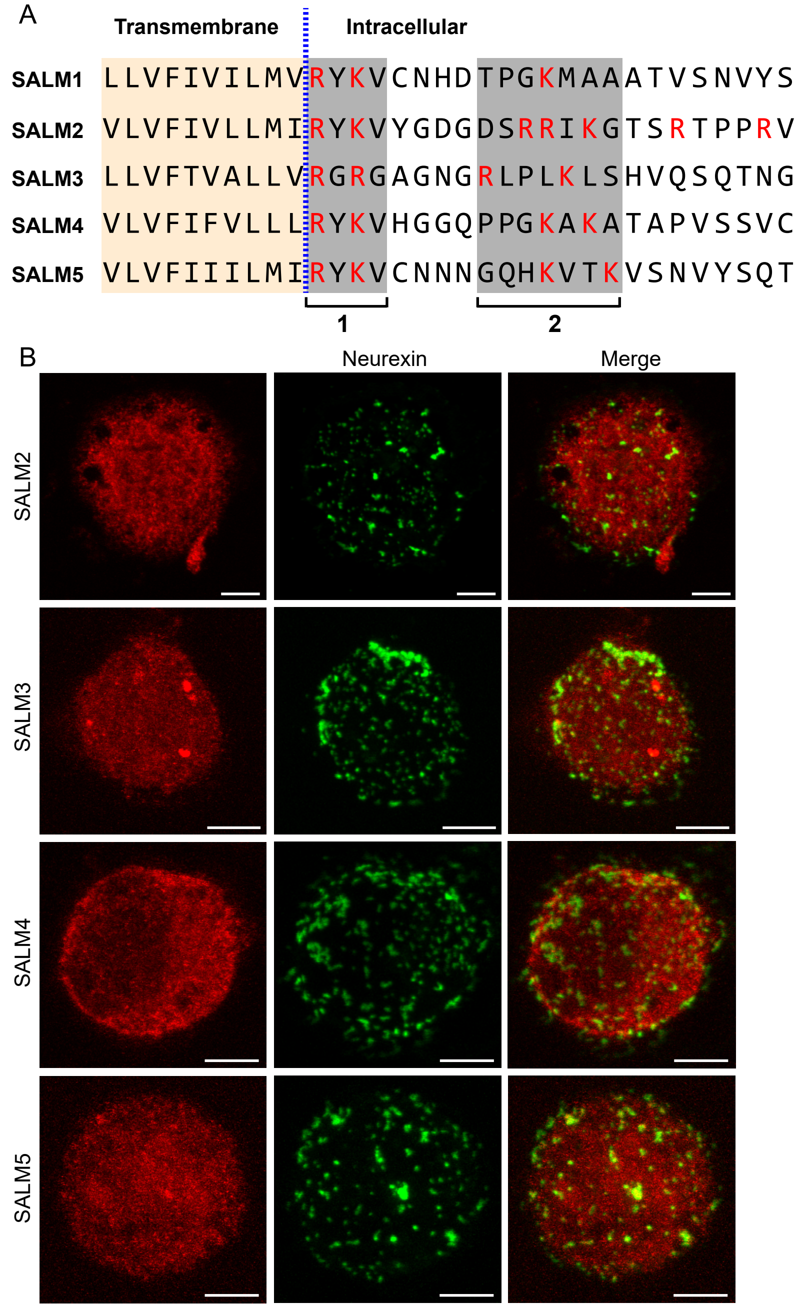

Figure S6. SALMs contain a highly conserved polybasic region and can all cluster Nrxn1β (Related to figure 5).

A) Partial amino acid sequences of the transmembrane and juxtamembrane domains of SALM1, 2, 3, 4 and 5. Positively charged amino acids in the polybasic regions are highlighted in red.

B) Examples of surface expression pattern of Nxn1β(-SS4)-pHI (green) in HEK cells when co-transfected with FLAG-SALM2, FLAG-SALM3, FLAG-SALM4 or FLAG-SALM5 (red, total staining). SALM2, 3, 4 and 5 were intracellularly stained for FLAG. Images represent collapsed z-stacks. Bars=5μm.

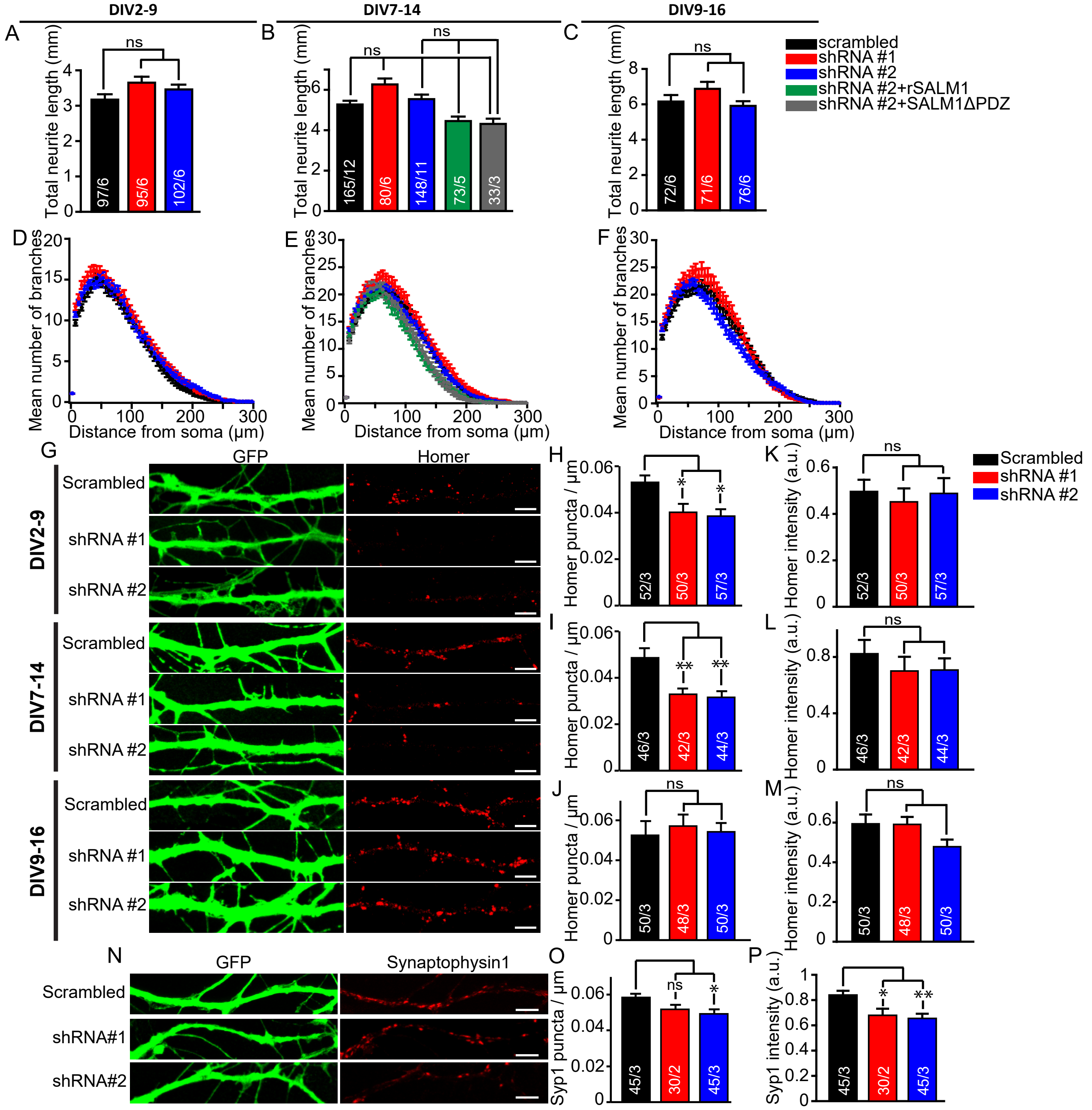

Figure S7. Knockdown of SALM1 does not affect neurite morphology, but impairs synapse development and synaptic vesicle recruitment (related to figure 5).

A-C) Mean total dendrite length ± SEM of autaptic hippocampal mouse neurons infected with SALM1 shRNAs and/or rescue plasmids at DIV2→9 (A), DIV7→14 (B) or DIV9→16 (C).

D-F) Sholl analysis of autaptic hippocampal neurons for DIV2→9 (D), DIV7→14 (E) or DIV9→16 (F). Data points indicate mean number of branches ± SEM for given distances from the soma. The n numbers are the same for each condition as indicated in A-C.

G) Example images of neurites from mouse autaptic excitatory hippocampal neurons stained for GFP and Homer. Cells were infected with viruses carrying the scrambled or the SALM1 shRNA constructs at three different time points (DIV2, DIV7 or DIV9) and were analyzed seven days later (DIV9, DIV14 or DIV16). Bars=5µm.

H-J) Average number of Homer puncta per µm neurite ± SEM for DIV2→9 (H), DIV7→14 (I) and DIV9→16 (J).

K-M) Average intensity ± SEM of Homer puncta for DIV2→9 (K), DIV7→14 (L) and DIV9→16 (M).

N-P are described on page 10

Figure S7. Continued

N) Example images of neurites from mouse autaptic hippocampal neurons stained for GFP and Synaptophysin1. Cells were infected with viruses carrying the scrambled or the SALM1 shRNA constructs at DIV7 and were analyzed at DIV14. Bars=5μm.

O) Average number of Synaptophysin1 puncta per μm neurite ± SEM for DIV7→14.

P) Average intensity ± SEM of Synaptophysin1 puncta.

For all bar graphs, the numbers given at the base of the bars indicate the total number of neurons used / the total number of independent cultures used. Kruskal-Wallis tests with post hoc paired comparisons were used on all data sets. ns=not significant, *=$p<0.05$, **=$p<0.01$ and ***=$p<0.001$.

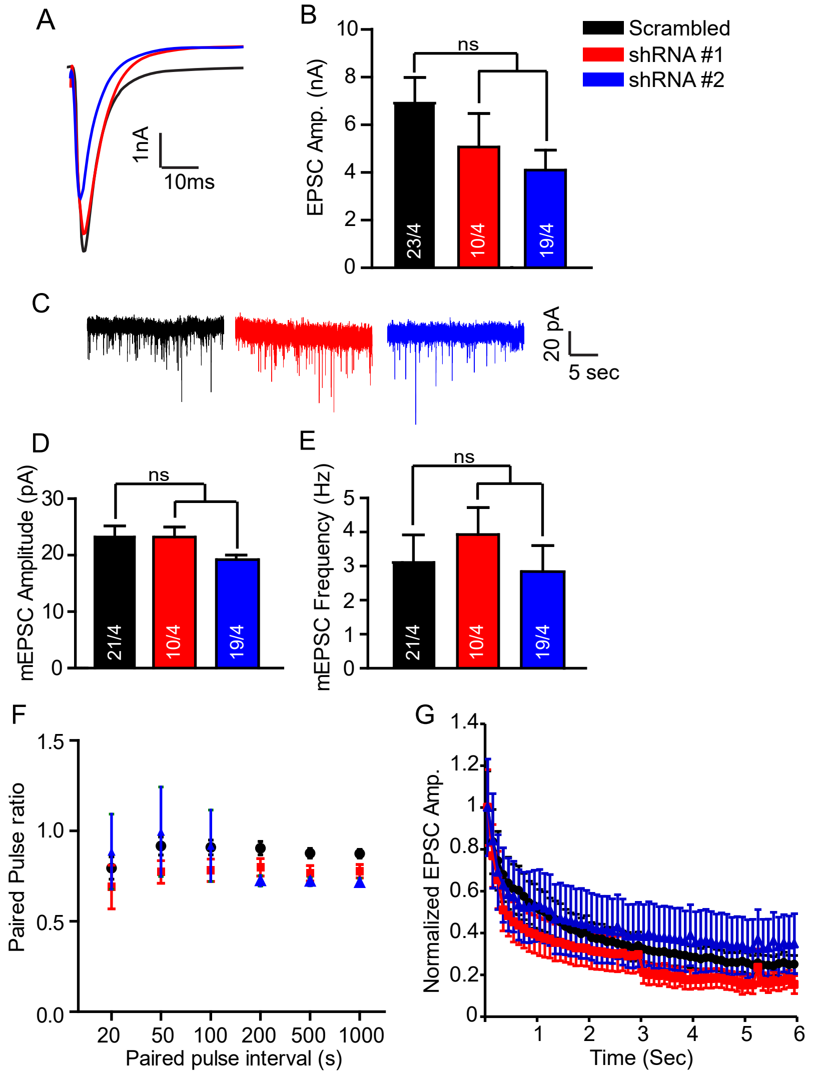

Figure S8. SALM1 depletion does not affect synaptic transmission during late developmental stage (DIV9→16) (related to figure 6).

A) Example traces of EPSCs in control (black), shRNA#1 (red), shRNA#2 (blue) or shRNA#2+SALM1 (green) expressing autaptic mouse hippocampal neurons.

B) Average evoked EPSC amplitudes ± SEM. Numbers at the base of the bars indicate the total number of autaptic neurons observed / total number of independent cultures used.

C) Example traces of mEPSCs from control (black), shRNA#1 (red) or shRNA#2 (blue) expressing autaptic mouse hippocampal neurons.

D-E) Average mEPSC amplitudes ± SEM (D) or average frequencies ± SEM (E). Numbers at the base of the bars indicate the total number of autaptic neurons observed / total number of independent cultures used.

F) Average paired pulse ratios obtained in autaptic mouse hippocampal neurons using different paired pulse intervals ± SEM. N=18 n=11 and n=13 neurons for scrambled, shRNA#1 and shRNA#2 respectively.

G) Average normalized EPSC response ± SEM of autaptic mouse hippocampal neurons upon 10Hz train stimulation. N=20, n=13 and n=15 neurons for scrambled, shRNA#1 and shRNA#2 respectively.

References

Bubb MR, Senderowicz AM, Sausville EA, Duncan KL, Korn ED. 1994. Jasplakinolide, a cytotoxic natural product, induces actin polymerization and competitively inhibits the binding of phalloidin to F-actin. J Biol Chem 269:14869-14871.
