# Supplementary material for: SALM1 controls synapse development by promoting F‐actin/PIP2‐dependent Neurexin clustering
Source: EMBO J. 2019 Aug 1;38(17):e101289. doi: 10.15252/embj.2018101289 (PMC6717895; doi:10.15252/embj.2018101289)
Supplement: Supplementary file 2 — Expanded View Figures PDF [file EMBJ-38-e101289-s002.pdf]

Expanded View Figures

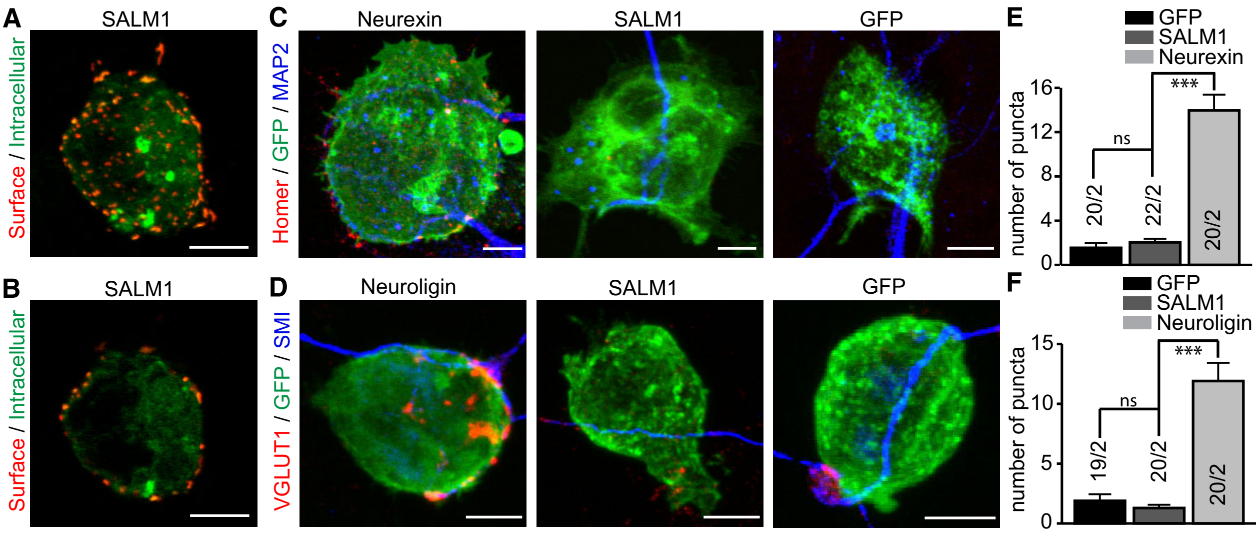

Figure EV1. SALM1 does not induce synaptogenesis in mixed culture assays.

A Collapsed *z*-stack image of a calcium phosphate transfected HEK cell showing surface expression (red) and intracellular expression (green) of SALM1-pHl.

B Single *z*-slice from the *z*-stack image given in (A).

C, D Example images of calcium phosphate transfected HEK cells expressing GFP, SALM1-pHl, Nrxn1β-pHl, or HA-Nlg1 (green) co-cultured with DIV9-10 sandwich-cultured mouse hippocampal neurons. Dendrites were stained for MAP2 (blue), postsynapses for Homer (C), and presynapses for VGluT1 (D) (red).

E, F Average number of postsynapses ± SEM (E) or presynapses ± SEM (F) per HEK cell. Numbers in bars indicate total number of cells/total number of independent cultures. Kruskal–Wallis tests with post hoc pairwise comparisons were used for (E) ($P < 0.001$) and (F) ($P < 0.001$). ns = not significant and $^{***}P < 0.001$.

Data information: Scale bars (A–D) = 5 µm.

Source data are available online for this figure.

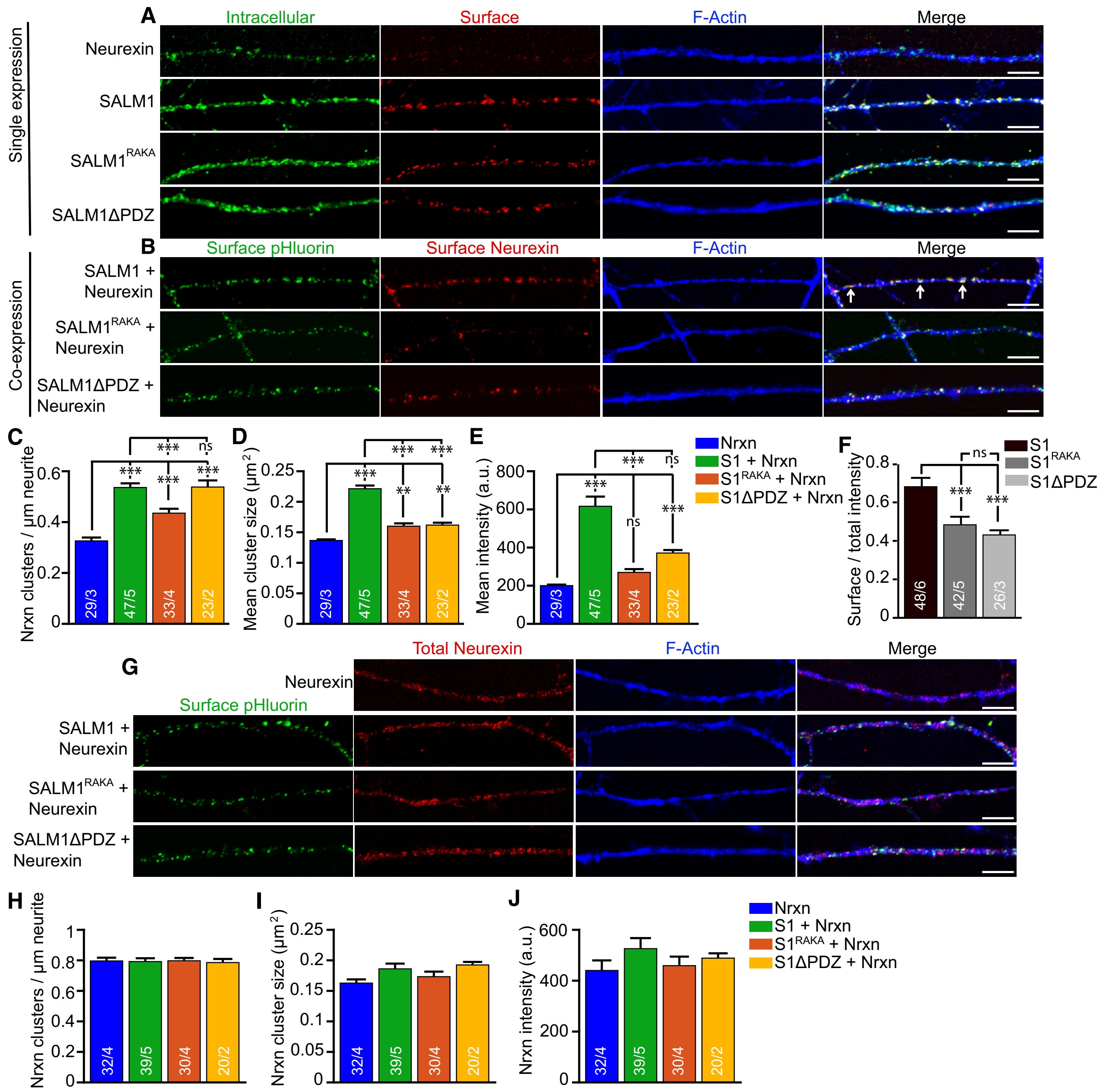

Figure EV2.

 © 2019 The Authors

Figure EV2. SALM1 mediates Nrxn1β expression on the surface of hippocampal neurons.

A Example images of neurites from DIV 11 sandwich-cultured mouse excitatory hippocampal neurons lentivirally infected at DIV4 with Nrxn1β-FLAG, SALM1-pHl, or SALM1^{RAKA}-pHl. Intracellular and surface stainings are shown for each construct. Bars = 5 μm.

B Example images of neurites from DIV 11 sandwich-cultured mouse excitatory hippocampal neurons lentivirally co-infected at DIV4 with Nrxn1β-FLAG and SALM1-pHl or Nrxn1β-FLAG and SALM1^{RAKA}-pHl. Surface stainings are shown for each construct. Arrows indicate examples of overlap between SALM1-pHl and Nrxn1β-FLAG clusters. Bars = 5 μm.

C–E Average number (C), size (D), and intensity (E) ± SEM of surface Nrxn1β-FLAG clusters.

F Average ratio ± SEM of surface over total staining intensity for SALM1-pHl and SALM1^{RAKA}-pHl.

G Example images of neurites from DIV 11 sandwich-cultured mouse excitatory hippocampal neurons lentivirally co-infected at DIV4 with Nrxn1β-FLAG and SALM1-pHl or Nrxn1β-FLAG and SALM1^{RAKA}-pHl. Surface stainings are shown for SALM1-pHl and SALM1^{RAKA}-pHl. Total staining is shown for Nrxn1β-FLAG. Bars = 5 μm.

H–J Average number (H), size (I), and intensity (J) ± SEM of total (intracellular and surface) Nrxn1β-FLAG clusters.

Data information: For all graphs, the *n* is indicated in the bars and represents the total number of cells/total number of independent cultures. A one-way ANOVA test with post hoc Bonferroni test was used for (C) ($P < 0.001$), (F) ($P < 0.001$), and (H) ($P = 0.987$). Kruskal–Wallis tests with post hoc paired comparisons were used for (D) ($P < 0.001$), (E) ($P < 0.001$), (I) ($P = 0.06$), and (J) ($P = 0.192$). ns = not significant, ** $P < 0.01$, and *** $P < 0.001$.

Source data are available online for this figure.

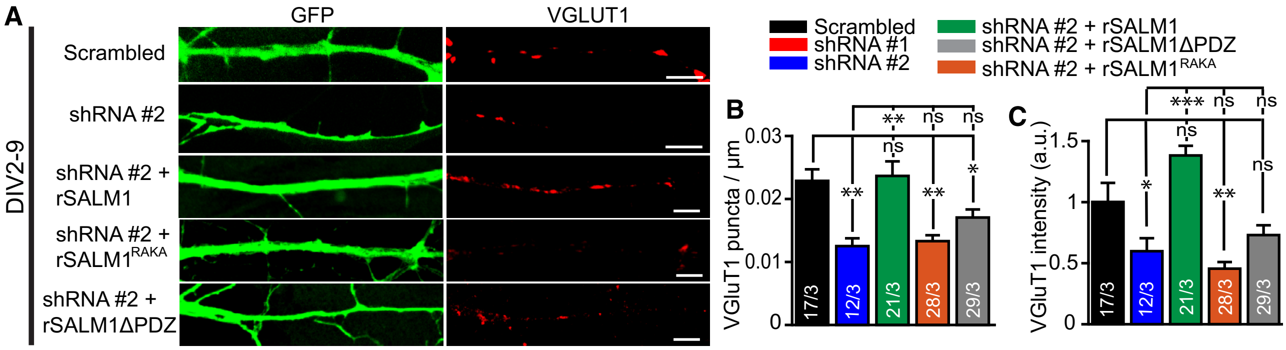

Figure EV3. Knockdown of SALM1 decreases synapse formation and synaptic vesicle clustering during early development.

A Example images of neurites from single isolated (autaptic) mouse excitatory hippocampal neurons stained for GFP and VGluT1. Cells were lentivirally infected with scrambled, shRNA#2, shRNA#2 + rSALM1 shRNA#2 + rSALM1^{RAKA} or with shRNA#2 + rSALM1ΔPDZ at DIV2 and analyzed 7 days later (DIV9). Bars = 5 μm.

B Average number of VGluT1 puncta per μm neurite ± SEM per neuron (Kruskal–Wallis, $P < 0.001$).

C Average intensity ± SEM of VGluT1 puncta per neuron (Kruskal–Wallis, $P < 0.001$).

Data information: For all graphs, numbers in bars indicate total number of neurons/total number of independent cultures. ns = not significant, * $P < 0.05$, ** $P < 0.01$, and *** $P < 0.001$.

Source data are available online for this figure.
